# The oral health care needs of people living in residential aged care, Australia, 2016–20: a retrospective cross‐sectional study

**DOI:** 10.5694/mja2.52625

**Published:** 2025-03-16

**Authors:** Gillian E Caughey, Tracy Air, Miia Rahja, Maria C Inacio

**Affiliations:** ^1^ Registry of Senior Australians (ROSA) Research Centre South Australian Health and Medical Research Institute Adelaide SA; ^2^ Registry of Senior Australians (ROSA) Research Centre Caring Futures Institute, Flinders University Adelaide SA; ^3^ Flinders Health and Medical Research Institute Flinders University Adelaide SA

**Keywords:** Aged, Health services for the aged, Dental health services, Health services research

Providing high quality medical care for people living in residential aged care is a national challenge, and oral health care is one area that is inadequate.^1^ In 2014, 84.7% of residents in Victorian nursing homes had untreated dental decay;^2^ in 2015, 53% of Australians aged 65 years or older had periodontal disease and 19% complete tooth loss.^3^ Poor oral health is associated with other adverse health outcomes, including cardiovascular disease, cognitive decline, and pneumonia.^4^ The Royal Commission into Aged Care Quality and Safety recommended that the access of aged care home residents to oral health practitioners be improved.^1^ Apart from establishing the national Senior Dental Benefits Scheme and adding dental and oral health care to the Quality of Care Principles, evidence of effective action has, however, been limited.^5^

We therefore estimated the incidence of oral and dental‐related care needs and health service and medication use for aged care residents, analysing data for the national historical cohort of the Registry of Senior Australians (ROSA),^6^ 1 July 2016 – 30 June 2020. We included all 360 305 non‐Indigenous residents of aged care homes aged 65 years or older who did not hold Department of Veterans’ Affairs (DVA) concession cards (access to Medicare Benefits Schedule [MBS] items is different for DVA card holders). Oral health information for 137 113 residents was based on the aged care eligibility assessments that have been conducted by the aged care eligibility assessment team since 1 July 2017. The assessment includes a mandatory question about problems with teeth, mouth, or dentures, including tooth loss, dental cavities, periodontal disease, and gingivitis (inflammation of the gums, dry mouth, tooth wear). We estimated crude and direct age‐ and sex‐standardised (reference year: 2017–18 ROSA study cohort) cumulative incidence of hospitalisations with oral health or dental‐related diagnoses as proportions with 95% confidence intervals (CIs), dental practitioner health service use (MBS data), and the dispensing of medications prescribed by public and private dentists (Pharmaceutical Benefits Scheme Dental Schedule data), both overall and by financial year (Supporting Information, table 1). Hospitalisation analyses were limited by the availability of ROSA historical cohort data to aged care residents admitted to public hospitals in South Australia, New South Wales, and Victoria (private hospitals data are not available for South Australia). The study was approved by the University of South Australia human research ethics committee (200489), the Australian Institute of Health and Welfare ethics committee (EO2022/4/1376), the South Australian Department for Health and Wellbeing human research ethics committee (HREC/18/SAH/90), and the New South Wales Population and Health Services research ethics committee (2019/ETH12028).

The median age of the 360 305 eligible residents in 2830 residential aged care homes was 85 years (interquartile range [IQR], 80–90 years); 226 490 were women (62.9%), 192 310 were living with dementia (53.4%), and they had a median five (IQR, 3–7) health conditions. Median study follow‐up time for residents was 499 days (IQR, 188–1002 days). Oral health care problems were identified for 26 842 of 137 113 people (19.6%; 95% CI, 19.4–19.8%). Of all eligible residents, 665 people (0.18%; 95% CI, 0.17–0.20%) had used MBS‐subsidised dental practitioner health services, and 6605 (1.83%; 95% CI, 1.79–1.88%) had used medications prescribed by dentists (Box). A total of 4954 aged care residents in South Australia, New South Wales, and Victoria (1.99%; 95% CI, 1.94–2.05%) had been hospitalised with dental or oral‐related diagnoses (Supporting Information, table 3); 1167 hospitalisations (0.47% of residents; 95% CI, 0.44–0.50% were potentially preventable (Box).

Box 1 Age‐ and sex‐standardised cumulative incidence of oral health and dental‐related health service, medications, and hospital use by aged care residents included in the national historical cohort of the Registry of Senior Australians (ROSA), 1 July 2016 – 30 June 2020, overall and by study year*

**Table:** 

Characteristic	2016–17 to 2019–20	2016–17	2017–18	2018–19	2019–20
**All aged care residents**	360 305	198 734	202 878	206 001	210 590
**Medicare Benefits Scheme‐subsidised dental practitioner health service**					
Dental practitioner health services	665	182	182	192	142
Cumulative incidence (95% CI)	0.18% (0.17–0.20%)	0.09% (0.08–0.11%)	0.09% (0.08–0.10%)	0.09% (0.08–0.11%)	0.07% (0.06–0.08%)
**Pharmaceutical Benefits Scheme Dental Schedule medications**					
Antibacterial medications (systemic)	5988	1691	1686	1739	1428
Cumulative incidence (95% CI)	1.66% (1.62–1.70%)	0.85% (0.81–0.89%)	0.83% (0.79–0.87%)	0.84% (0.81–0.88%)	0.68% (0.64–0.71%)
Anti‐inflammatory and anti‐rheumatic medications	43	13	13	9	9
Cumulative incidence (95% CI)	0.01% (0.01–0.02%)	0.01% (0.00–0.01%)	0.01% (0.00–0.01%)	0.004% (0.00–0.01%)	0.004% (0.00–0.01%)
Analgesic medications	574	143	160	151	137
Cumulative incidence (95% CI)	0.16% (0.15–0.17%)	0.07 (0.06–0.08%)	0.08% (0.07–0.09%)	0.07% (0.06–0.09%)	0.07% (0.05–0.08%)
**New South Wales, Victoria, South Australia aged care residents**	248 684	138 694	141 144	142 591	144 695
**Oral/dental health‐related hospitalisations**					
Any dental hospitalisation (primary or secondary diagnosis)	4954	1251	1289	1328	1261
Cumulative incidence (95% CI)	1.99% (1.94–2.05%)	0.90% (0.85–0.95%)	0.91% (0.86–0.96%)	0.93% (0.88–0.98%)	0.87% (0.82–0.92%)
Potentially preventable dental hospitalisation (primary or secondary diagnosis)	1167	304	287	313	282
Cumulative incidence (95% CI)	0.47% (0.44–0.50%)	0.22% (0.19–0.24%)	0.20% (0.18–0.23%)	0.22% (0.20–0.25%)	0.20% (0.17–0.22%)
Potentially preventable dental hospitalisation (primary diagnosis only)	380	101	88	116	82
Cumulative incidence (95% CI)	0.15% (0.14–0.17%)	0.07% (0.06–0.09%)	0.06% (0.05–0.08%)	0.08% (0.07–0.10%)	0.06% (0.04–0.07%)
Potentially preventable dental emergency department presentations	353	72	88	105	90
Cumulative incidence (95% CI)	0.14% (0.13–0.16%)	0.05% (0.04–0.06%)	0.06% (0.05–0.08%)	0.07% (0.06–0.09%)	0.06% (0.05–0.08%)
Dental extractions and restoration	191	47	47	57	43
Cumulative incidence (95% CI)	0.08% (0.07–0.09%)	0.03% (0.03–0.04%)	0.03% (0.02–0.04%)	0.04% (0.03–0.05%)	0.03% (0.02–0.04%)
Oral and dental disorders	795	202	189	235	186
Cumulative incidence (95% CI)	0.32% (0.30–0.34%)	0.15% (0.13–0.17%)	0.13% (0.12–0.15%)	0.17% (0.14–0.19%)	0.13% (0.11–0.15%)

CI = confidence interval.

* The crude values are provided in the Supporting Information, table 2; the results are presented as rates per 1000 residents in the Supporting Information, table 3.

Our findings, based on a population‐based evaluation, can be generalised to all aged care home residents in Australia. However, we could not include dental services provided through the residential aged care home or paid for privately (ie, services that were not MBS‐subsidised); data for private dental care in aged care homes is required to assess whether dental and oral health care needs are being met. In March 2024, 54.9% of older Australians had private health insurance,^7^ which can include subsidisation of private dental services.

Despite recent government initiatives to improve the oral health of older Australians, including the *National Oral Health Plan 2015–2024*,^8^ current models of care and service delivery in residential aged care are not meeting the needs of residents. The system, service, and workforce barriers to improving oral health include high staff turnover and the lack of oral health education for staff, the high costs and access problems of dental and oral health services, and the inadequate integration of aged care and health care systems.^9^, ^10^ Encouragingly, the publicly funded dental domiciliary service program of the Sydney Local Health District, the Inner West Oral Health Outreach Program (Reach‐OHT), has shown that a multidisciplinary team providing oral health assessments and treatments (largely diagnostic and preventive services) is a feasible and sustainable approach to oral health care for aged care residents.^11^ Oral health policy and practice reforms are urgently needed to improve the health and wellbeing of older Australians living in aged care homes.

## Open access

Open access publishing facilitated by Flinders University, as part of the Wiley – Flinders University agreement via the Council of Australian University Librarians.

## Competing interests

No relevant disclosures.

## Data sharing

The data underlying this report are not available for sharing because of restrictions imposed by the ethics and original data custodian approval.

## Supporting information


Supplementary methods and results
