# Supplementary material for: The oral health care needs of people living in residential aged care, Australia, 2016–20: a retrospective cross‐sectional study
Source: Med J Aust. 2025 Mar 16;222(6):318–20. doi: 10.5694/mja2.52625 (PMC11972596; doi:10.5694/mja2.52625)
Supplement: Supplementary file 1 — Supplementary methods and results [file MJA2-222-318-s001.pdf]

MJA
The Medical Journal of Australia

Supporting Information

Supplementary methods and results

This appendix was part of the submitted manuscript and has been peer reviewed. It is posted as supplied by the authors.

Appendix to: Caughey GE, Air T, Rahja M, Inacio MC. The oral health care needs of people living in residential aged care, Australia, 2016–20: a retrospective cross-sectional study. *Med J Aust* 2025; doi: 10.5694/mja2.52625.

Table 1. Definitions and codes health service (Medicare Benefits Schedule), medications (Pharmaceutical Benefits Scheme, Dental Schedule), and hospitalisations

Description	Codes
**Health services (Medicare Benefits Schedule [MBS])**	MBS Item Code
Professional attendance (other than a second or subsequent attendance in a single course of treatment) by an approved dental practitioner, at consulting rooms, hospital or residential aged care facility where the patient is referred	51700
Professional attendance by an approved dental practitioner, each attendance subsequent to the first in a single course of treatment at consulting rooms, hospital or residential aged care facility where the patient is referred	51793
**Medications prescribed by dental practitioners (Pharmaceutical Benefits Scheme [PBS] Dental Schedule)**	PBS Item Codes
**Antibacterial medications for systemic use**	
Amoxicillin	3301R, 3393N, 3300Q, 5225B, 3310F, 3302T, 13184X
Amoxicillin and clavulanic acid	5011R, 13190F, 5009P, 5008N, 5006L
Benzathine benzylpenicillin	11735P, 5027N
Benzylpenicillin	3399X, 3398W
Cefaclor	5046N, 5047P, 5045M
Cefalexin	3317N, 3318P, 3319Q, 13278W, 3320R
Cefotaxime	1768C
Cefuroxime	11191B, 11228Y, 5052X
Clindamycin	5057E
Dicloxacillin	5096F, 5097G
Doxycycline	3321T, 5082L, 3322W
Erythromycin	3325B, 3334L, 3337P
Flucloxacillin	5257Q, 5258R, 5090X, 5091Y, 5095E
Lincomycin	11366F, 5144R
Metronidazole	5157K, 3339R, 5155H, 3341W
Phenoxymethylpenicillin	3363B, 3364C, 5012T, 5024K, 5029Q, 13291M, 3360W, 3361X
Procaine benzylpenicillin	3371K
Roxithromycin	5259T, 5250W, 5261X
Trimethoprim + sulfamethoxazole	3391L, 3390K
Vancomycin	5083M, 3323X
**Anti-inflammatory and antirheumatic medications**	
Diclofenac	5079H, 5076E, 5077F
Ibuprofen	5123P, 5124Q
Indometacin	5126T, 5128X
Ketoprofen	5136H
Naproxen	5179N, 5176K, 5178M, 5186Y
Piroxicam	5210R, 5204X, 5202T, 5203W
**Analgesic medications**	
Codeine	12054K, 5063L
Hydromorphone	12559B, 12045Y, 5115F, 12032G, 5116G, 12010D, 5117H
Morphine	10863R, 10858L, 12067D, 5163R, 5168B, 5169C, 5170D, 5239R, 5237P, 5238Q
Oxycodone	12311Y, 13234M, 5194J, 12074L, 5197M, 5191F, 5190E, 5195K
Paracetamol + codeine	12066C, 3316M

Description	Codes
Tramadol	12024W, 5232J, 5231H, 5150C
Codeine	12054K, 5063L
**Hospitalisations (International Classification of Diseases-10 Australian modification [ICD-10AM])**	ICD-10AM Codes
**Potentially preventable dental hospitalisations**	
Dental caries	K02*
Other diseases of hard tissues of teeth	K03*
Diseases of pulp and periapical tissues	K04*
Gingivitis and periodontal diseases	K05*
Other disorders of gingiva and edentulous alveolar ridge	K06 *
Other disorders of teeth and supporting structures	K08*
Other cysts of oral region, not elsewhere classified	K09.8
Cyst of oral region, unspecified	K09.9
Stomatitis and related lesions	K12*
Other diseases of lip and oral mucosa	K13*
Diseases of tongue	K14*
**Potentially preventable dental emergency department presentations**	
Dental caries	K02*
Other diseases of hard tissues of teeth	K03*
Diseases of pulp and periapical tissues	K04*
Gingivitis and periodontal diseases	K05*
Other disorders of gingiva and edentulous alveolar ridge	K06 *
Other disorders of teeth and supporting structures	K08*
Other cysts of oral region, not elsewhere classified	K09.8
Cyst of oral region, unspecified	K09.9
Stomatitis and related lesions	K12*
Other diseases of lip and oral mucosa	K13*
Diseases of tongue	K14*
**Dental procedures requiring anaesthesia, Australian Classification of Health Interventions (ACHI)**	ACHI codes
Nonsurgical removal of tooth	457*
Surgical removal of tooth	458*
Pulp treatment	462*
Periradicular surgery	463*
Metallic restoration	465*
Tooth-coloured restoration	466*
Other restorative dental service	469*
Crown	470*
Bridge	471*
Other dental service on crown and bridge	472*
Tooth root resection, per root	97241-00
Replantation and splinting of tooth	97387-00
Transplantation of tooth or tooth bud	97388-00
Exploration or negotiation of calcified root canal, per canal	97445-00
Obturation of resorption defect or perforation	97457-00
Interim therapeutic root filling	97458-00
Provision of resin splint, indirect	97772-00
Provision of metal splint, indirect	97773-00

Description	Codes
Metallic inlay for denture tooth	97778-00
**Australian refined diagnosis-related groups (AR-DRGs) D40 dental extractions and restorations (principal diagnosis (AD40PDX1)**	ICD-10AM codes
Nonsurgical removal of tooth	B00.2
Surgical removal of tooth	B37.0
Pulp treatment	D10.0
Periradicular surgery	D10.1
Metallic restoration	D10.2
Tooth-coloured restoration	D10.3
Other restorative dental service	D16.5
Crown	E10.63
Bridge	E11.63
Other dental service on crown and bridge	E13.63
Tooth root resection, per root	E14.63
Disorders of tooth development and eruption	K00*
Embedded and impacted teeth	K01*
Dental caries	K02*
Other diseases of hard tissues of teeth	K03*
Diseases of pulp and periapical tissues	K04*
Gingivitis and periodontal diseases	K05*
Gingival recession	K06.0
Gingival enlargement	K06.1
Gingival and edentulous alveolar ridge lesions associated with trauma	K06.2
Other specified disorders of gingiva and edentulous alveolar ridge	K06.8
Disorder of gingiva and edentulous alveolar ridge, unspecified	K06.9
Dentofacial anomalies	K07*
Exfoliation of teeth due to systemic causes	K08.0
Loss of teeth due to accident, extraction or local periodontal disease	K08.1
Atrophy of edentulous alveolar ridge	K08.2
Retained dental root	K08.3
Pathological fracture of tooth	K08.81
Other specified disorders of teeth and supporting structures	K08.88
Disorder of teeth and supporting structures, unspecified	K08.9
Cysts of oral region, not elsewhere classified	K09*
Other diseases of jaw	K10*
Stomatitis and related lesions	K12*
Other diseases of lip and oral mucosa	K13*
Diseases of tongue	K14*
Macrostomia	Q18.4
Microstomia	Q18.5
Macrocheilia	Q18.6
Microcheilia	Q18.7
Cleft hard palate	Q35.1*
Cleft soft palate	Q35.3*
Cleft uvula	Q35.7
Cleft palate, unspecified	Q35.9

Description	Codes
Congenital malformations of lips, not elsewhere classified	Q38.0*
Ankyloglossia	Q38.1*
Macroglossia	Q38.2*
Other congenital malformations of tongue	Q38.3*
Congenital malformations of salivary glands and ducts	Q38.4*
Congenital malformations of palate, not elsewhere classified	Q38.5*
Other congenital malformations of mouth	Q38.6*
Open wound of maxillary region	S01.42
Open wound of mandibular region	S01.43
Open wound of mouth, part unspecified	S01.50
Open wound of lip	S01.51
Open wound of buccal mucosa	S01.52
Open wound of gum (alveolar process)	S01.53
Open wound of tongue and floor of mouth	S01.54
Dislocation of tooth	S03.2
Sprain and strain of jaw	S03.4
Foreign body in mouth	T18.0
**AR-DRG D40 dental extractions and restorations intervention procedure (AP40INT1)**	ACHI codes
Tooth root resection, per root	97241-00
Surgical periodontal procedure, not elsewhere classified, per tooth or implant	97245-00
Nonsurgical removal of tooth	457*
Surgical removal of tooth	458*
Replantation and splinting of tooth	97387-00
Metallic restoration	465*
Tooth-coloured restoration	466*
Other restorative dental service	469*
Crown	470*
Bridge	471*
Other dental service on crown and bridge	472*
Fitting of implant abutment, per abutment	97661-00
Full crown attached to osseointegrated implant, non-metallic, indirect	97671-00
Full crown attached to osseointegrated implant, veneer, indirect	97672-00
Full crown attached to osseointegrated implant, metallic, indirect	97673-00
Provision of resin splint, indirect	97772-00
Provision of metal splint, indirect	97773-00
Metallic inlay for denture tooth	97778-00
**AR-DRG D67 oral and dental disorders (AD67PDX1)**	ICD-10AM codes
Nonsurgical removal of tooth	B00.2
Surgical removal of tooth	B37.0
Pulp treatment	D10.0
Periradicular surgery	D10.1
Metallic restoration	D10.2
Tooth-coloured restoration	D10.3
Other restorative dental service	D16.5
Crown	E10.63
Bridge	E11.63

Description	Codes
Other dental service on crown and bridge	E13.63
Tooth root resection, per root	E14.63
Disorders of tooth development and eruption	K00*
Embedded and impacted teeth	K01*
Dental caries	K02*
Other diseases of hard tissues of teeth	K03*
Diseases of pulp and periapical tissues	K04*
Gingivitis and periodontal diseases	K05*
Gingival recession	K06.0
Gingival enlargement	K06.1
Gingival and edentulous alveolar ridge lesions associated with trauma	K06.2
Other specified disorders of gingiva and edentulous alveolar ridge	K06.8
Disorder of gingiva and edentulous alveolar ridge, unspecified	K06.9
Dentofacial anomalies	K07*
Exfoliation of teeth due to systemic causes	K08.0
Loss of teeth due to accident, extraction or local periodontal disease	K08.1
Atrophy of edentulous alveolar ridge	K08.2
Retained dental root	K08.3
Pathological fracture of tooth	K08.81
Other specified disorders of teeth and supporting structures	K08.88
Disorder of teeth and supporting structures, unspecified	K08.9
Cysts of oral region, not elsewhere classified	K09*
Other diseases of jaw	K10*
Stomatitis and related lesions	K12*
Other diseases of lip and oral mucosa	K13*
Diseases of tongue	K14*
Macrostomia	Q18.4
Microstomia	Q18.5
Macrocheilia	Q18.6
Microcheilia	Q18.7
Cleft hard palate	Q35.1*
Cleft soft palate	Q35.3*
Cleft uvula	Q35.7
Cleft palate, unspecified	Q35.9
Cleft lip, bilateral	Q36.0
Cleft lip, median	Q36.1
Cleft lip, unilateral	Q36.9
Congenital malformations of lips, not elsewhere classified	Q38.0*
Ankyloglossia	Q38.1*
Macroglossia	Q38.2*
Other congenital malformations of tongue	Q38.3*
Congenital malformations of salivary glands and ducts	Q38.4*
Congenital malformations of palate, not elsewhere classified	Q38.5*
Other congenital malformations of mouth	Q38.6*
Open wound of maxillary region	S01.42
Open wound of mandibular region	S01.43

Description	Codes
Open wound of mouth, part unspecified	S01.50
Open wound of lip	S01.51
Open wound of buccal mucosa	S01.52
Open wound of gum (alveolar process)	S01.53
Open wound of tongue and floor of mouth	S01.54a
Dislocation of tooth	S03.2
Sprain and strain of jaw	S03.4
Foreign body in mouth	T18.0

ACHI, Australian Classification of Health Interventions; AR-DRG, Australian refined diagnosis-related groups; ED, emergency department; ICD-10AM, International Classification of Diseases-10 Australian modification; MBS, Medicare Benefits Schedule; PBS, Pharmaceutical Benefits Scheme.

* Includes all ICD-10-AM, ACHI codes available under this block.

Table 2. Crude cumulative incidence of oral health and dental-related health service, medications, and hospital use by aged care residents included in the national historical cohort of the Registry of Senior Australians (ROSA), 1 July 2016 – 30 June 2020, overall and by study year

Characteristic	2016–17 to 2019–20	2016–17	2017–18	2018–19	2019–20
**All aged care residents**	360,305	198,734	202,878	206,001	210,590
**Medicare Benefits Scheme-subsidised dental practitioner health service**					
Dental practitioner health services	665	182	182	192	142
Cumulative incidence (95% CI)	0.18% (0.17–0.20%)	0.09% (0.08–0.11%)	0.09% (0.08–0.10%)	0.09% (0.08–0.11%)	0.07% (0.06–0.08%)
**Pharmaceutical Benefits Scheme Dental Schedule medications**					
Antibacterial medications (systemic)	5988	1691	1686	1739	1428
Cumulative incidence (95% CI)	1.66% (1.62–1.70%)	0.85% (0.81–0.89%)	0.83% (0.79–0.87%)	0.84% (0.81–0.88%)	0.68% (0.64–0.71%)
Anti-inflammatory and anti-rheumatic medications	43	13	13	9	9
Cumulative incidence (95% CI)	0.01% (0.01–0.02%)	0.01% (0.00–0.01%)	0.01% (0.00–0.01%)	0.004% (0.00–0.01%)	0.004% (0.00–0.01%)
Analgesic medications	574	143	160	151	137
Cumulative incidence (95% CI)	0.16% (0.15–0.17%)	0.07 (0.06–0.08%)	0.08% (0.07–0.09%)	0.07% (0.06–0.09%)	0.07% (0.05–0.08%)
**New South Wales, Victoria, South Australia aged care residents**	248 684	138 694	141 144	142 591	144 695
**Oral/dental health-related hospitalisations**					
Any dental hospitalisation (primary or secondary diagnosis)	4954	1251	1289	1328	1261
Cumulative incidence (95% CI)	1.99% (1.94–2.05%)	0.90% (0.85–0.95%)	0.91% (0.86–0.96%)	0.93% (0.88–0.98%)	0.87% (0.82–0.92%)
Potentially preventable dental hospitalisation (primary or secondary diagnosis)	1167	304	287	313	282
Cumulative incidence (95% CI)	0.47% (0.44–0.50%)	0.22% (0.20–0.24%)	0.20% (0.18–0.23%)	0.22% (0.20–0.25%)	0.19% (0.17–0.22%)
Potentially preventable dental hospitalisation (primary diagnosis only)	380	101	88	116	82
Cumulative incidence (95% CI)	0.15% (0.14–0.17%)	0.07% (0.06–0.09%)	0.06% (0.05–0.08%)	0.08% (0.07–0.10%)	0.06% (0.05–0.07%)
Potentially preventable dental emergency department presentations	353	72	88	105	90
Cumulative incidence (95% CI)	0.14% (0.13–0.16%)	0.05% (0.04–0.07%)	0.06% (0.05–0.08%)	0.07% (0.06–0.09%)	0.06% (0.05–0.08%)
Dental extractions and restoration	191	47	47	57	43
Cumulative incidence (95% CI)	0.08% (0.07–0.09%)	0.03% (0.03–0.05%)	0.03% (0.02–0.04%)	0.04% (0.03–0.05%)	0.03% (0.02–0.04%)
Oral and dental disorders	795	202	189	235	186
Cumulative incidence (95% CI)	0.32% (0.30–0.34%)	0.15% (0.13–0.17%)	0.13% (0.12–0.15%)	0.16% (0.15–0.19%)	0.13% (0.11–0.15%)

CI = confidence interval.

Table 3. Age- and sex-standardised rates of oral and dental hospitalisations per 1000 aged care residents, overall and by study year (New South Wales, Victoria, South Australia only)

Characteristic	Total	2016-17	2017-18	2018-19	2019-20
**Number of residents**	248,684	138,694	141,144	142,591	144,695
**Oral/dental-related hospitalisations**					
Any dental/oral related hospitalisation (primary and secondary diagnosis)	4954	1251	1289	1328	1261
Rate per 1000 residents (95% CI)	19.9 (19.4-20.5)	9.02 (8.26-9.50)	9.13 (8.63-9.63)	9.32 (8.82-9.82)	8.72 (8.24-9.21)
Potentially preventable dental hospitalisation (primary and secondary diagnosis)	1167	304	287	313	282
Rate per 1000 residents (95% CI)	4.69 (4.43-4.97)	2.19 (1.94-2.43)	2.03 (1.80-2.27)	2.20 (1.96-2.45)	1.95 (1.73-2.18)
Potentially preventable dental hospitalisation (primary diagnosis only)	380	101	88	116	82
Rate per 1000 residents (95% CI)	1.53 (1.38-1.69)	0.73 (0.58-0.87)	0.62 (0.49-0.75)	0.82 (0.67-0.97)	0.57 (0.45-0.69)
Potentially preventable dental emergency department presentations	353	72	88	105	90
Rate per 1000 residents (95% CI)	1.42 (1.28-1.58)	0.52 (0.40-0.64)	0.62 (0.43-0.75)	0.73 (0.59-0.87)	0.62 (0.50-0.75)
Dental extractions and restoration	191	47	47	57	43
Rate per 1000 residents (95% CI)	0.77 (0.67-0.88)	0.34 (0.24-0.44)	0.33 (0.23-0.43)	0.40 (0.30-0.51)	0.30 (0.21-0.39)
Oral and dental disorders	795	202	189	235	186
Rate per 1000 residents (95% CI)	3.19 (2.98-3.43)	1.45 (1.25-1.65)	1.34 (1.15-1.53)	1.66 (1.44-1.87)	1.29 (1.11-1.48)

STROBE Statement—Checklist of items that should be included in reports of *cross-sectional studies.* Note: The page numbers refer to the submitted manuscript, not to the published article or its supplementary file.

	Item No	Recommendation	Page No
**Title and abstract**	1	(*a*) Indicate the study's design with a commonly used term in the title or the abstract	1
		(*b*) Provide in the abstract an informative and balanced summary of what was done and what was found	1
**Introduction**			
Background/rationale	2	Explain the scientific background and rationale for the investigation being reported	2
Objectives	3	State specific objectives, including any prespecified hypotheses	2
**Methods**			
Study design	4	Present key elements of study design early in the paper	2
Setting	5	Describe the setting, locations, and relevant dates, including periods of recruitment, exposure, follow-up, and data collection	2
Participants	6	(*a*) Give the eligibility criteria, and the sources and methods of selection of participants	2
Variables	7	Clearly define all outcomes, exposures, predictors, potential confounders, and effect modifiers. Give diagnostic criteria, if applicable	2, Table S1
Data sources/ measurement	8*	For each variable of interest, give sources of data and details of methods of assessment (measurement). Describe comparability of assessment methods if there is more than one group	2,Table S1
Bias	9	Describe any efforts to address potential sources of bias	N/A
Study size	10	Explain how the study size was arrived at	N/A
Quantitative variables	11	Explain how quantitative variables were handled in the analyses. If applicable, describe which groupings were chosen and why	2,Table S1
Statistical methods	12	(*a*) Describe all statistical methods, including those used to control for confounding	2
		(*b*) Describe any methods used to examine subgroups and interactions	N/A
		(*c*) Explain how missing data were addressed	N/A
		(*d*) If applicable, describe analytical methods taking account of sampling strategy	N/A
		(*e*) Describe any sensitivity analyses	N/A
**Results**			
Participants	13*	(a) Report numbers of individuals at each stage of study—eg numbers potentially eligible, examined for eligibility, confirmed eligible, included in the study, completing follow-up, and analysed	N/A
		(b) Give reasons for non-participation at each stage	N/A
		(c) Consider use of a flow diagram	N/A
Descriptive data	14*	(a) Give characteristics of study participants (eg demographic, clinical, social) and information on exposures and potential confounders	2
		(b) Indicate number of participants with missing data for each variable of interest	N/A
Outcome data	15*	Report numbers of outcome events or summary measures	3,Table 1, Table S2, Table S3

Main results	16	(*a*) Give unadjusted estimates and, if applicable, confounder-adjusted estimates and their precision (eg, 95% confidence interval). Make clear which confounders were adjusted for and why they were included	Table 1, Table S2, Table S3
		(*b*) Report category boundaries when continuous variables were categorized	N/A
		(*c*) If relevant, consider translating estimates of relative risk into absolute risk for a meaningful time period	N/A
Other analyses	17	Report other analyses done—eg analyses of subgroups and interactions, and sensitivity analyses	N/A
**Discussion**			
Key results	18	Summarise key results with reference to study objectives	3
Limitations	19	Discuss limitations of the study, taking into account sources of potential bias or imprecision. Discuss both direction and magnitude of any potential bias	3
Interpretation	20	Give a cautious overall interpretation of results considering objectives, limitations, multiplicity of analyses, results from similar studies, and other relevant evidence	3
Generalisability	21	Discuss the generalisability (external validity) of the study results	3
**Other information**			
Funding	22	Give the source of funding and the role of the funders for the present study and, if applicable, for the original study on which the present article is based	Pending

*Give information separately for exposed and unexposed groups.

Note: An Explanation and Elaboration article discusses each checklist item and gives methodological background and published examples of transparent reporting. The STROBE checklist is best used in conjunction with this article (freely available on the Web sites of PLoS Medicine at http://www.plosmedicine.org/, Annals of Internal Medicine at http://www.annals.org/, and Epidemiology at http://www.epidem.com/). Information on the STROBE Initiative is available at www.strobe-statement.org.
